# Histone demethylase KDM1A promotes hepatic steatosis and inflammation by increasing chromatin accessibility in NAFLD

**DOI:** 10.1016/j.jlr.2024.100513

**Published:** 2024-01-29

**Authors:** Zifeng Yang, Siyao Zhang, Xiang Liu, Rui Shu, Wei Shi, Weiyi Qu, Dianyu Liu, Zhiwei Cai, Ye Wang, Xu Cheng, Yemao Liu, Xiao-Jing Zhang, Lan Bai, Hongliang Li, Zhi-Gang She

**Affiliations:** 1 Department of Cardiology, Renmin Hospital of Wuhan University, Wuhan, China; 2 Institute of Model Animal, Wuhan University, Wuhan, China; 3 Gannan Innovation and Translational Medicine Research Institute, State Key Laboratory of New Targets Discovery and Drug Development for Major Diseases, Gannan Medical University, Ganzhou, China; 4 School of Basic Medical Sciences, Wuhan University, Wuhan, China; 5 Department of Cardiology, Zhongnan Hospital of Wuhan University, Wuhan, China; 6 Department of Cardiology, Huanggang Central Hospital, Huanggang, China

**Keywords:** NAFLD, KDM1A, chromatin accessibility, lipid accumulation, inflammation

## Abstract

Nonalcoholic fatty liver disease (NAFLD) is the most prevalent chronic liver disease without specific Food and Drug Administration-approved drugs. Recent advances suggest that chromatin remodeling and epigenetic alteration contribute to the development of NAFLD. The functions of the corresponding molecular modulator in NAFLD, however, are still elusive. *KDM1A,* commonly known as lysine-specific histone demethylase 1, has been reported to increase glucose uptake in hepatocellular carcinoma. In addition, a recent study suggests that inhibition of *KDM1A* reduces lipid accumulation in primary brown adipocytes. We here investigated the role of *KDM1A*, one of the most important histone demethylases, in NAFLD. In this study, we observed a significant upregulation of *KDM1A* in NAFLD mice, monkeys, and humans compared to the control group. Based on these results, we further found that the *KDM1A* can exacerbate lipid accumulation and inflammation in hepatocytes and mice. Mechanistically, *KDM1A* exerted its effects by elevating chromatin accessibility, subsequently promoting the development of NAFLD. Furthermore, the mutation of *KDM1A* blunted its capability to promote the development of NAFLD. In summary, our study discovered that *KDM1A* exacerbates hepatic steatosis and inflammation in NAFLD via increasing chromatin accessibility, further indicating the importance of harnessing chromatin remodeling and epigenetic alteration in combating NAFLD. *KDM1A* might be considered as a potential therapeutic target in this regard.

Nonalcoholic fatty liver disease (NAFLD) is a hepatic disorder characterized by the excessive accumulation of hepatic fat in the absence of excessive alcohol consumption or other liver diseases (1, 2). It has emerged as one of the most prevalent chronic liver conditions, affecting approximately 25.24% of the global population (3, 4). More than 25% of NAFLD patients progress to nonalcoholic steatohepatitis (NASH), a condition associated with a significantly heightened risk of hepatocellular carcinoma and extrahepatic cardiovascular disorders (4, 5, 6). NAFLD is becoming a prominent underlying factor necessitating liver transplantation in cases of advanced liver disease (7, 8). However, there is currently no specific drug approved by the Food and Drug Administration for NAFLD. Consequently, there is an urgent need for therapeutic targets and viable therapeutic strategies for NAFLD.

Chromatin remodeling and epigenetic modifications following metabolic stress play a vital role in the pathogenesis of NAFLD (9, 10, 11). Among these modifications, histone methylation, which is tightly regulated by various epigenetic enzymes, has gained significant attention due to its essential role in the progression of NAFLD (9, 12, 13). The functions of the corresponding molecular modulator in NAFLD, however, are still elusive. *KDM1A*, known as lysine-specific histone demethylase1, specifically removes methylation of H3K4me or H3K9me, causing gene expression repression and activation, respectively (14, 15). *KDM1A* has been reported to increase the expression levels of inflammatory response genes by acting as a positive regulator in inflammatory diseases such as hepatitis B virus–associated glomerulonephritis and rheumatoid (16, 17). *Kdm1a* knockdown decreases lipid accumulation in primary brown adipocytes (18). *KDM1A* has also recently been recognized as a pivotal epigenetic modulator suppressing hepatic mitochondrial respiration (19). However, the functions of *KDM1A* in NAFLD remain unclear.

In this study, we observed a remarkable upregulation of *KDM1A* in NAFLD and proved that *KDM1A* exacerbates hepatic steatosis and inflammation in NAFLD via increasing chromatin accessibility. These findings indicated the importance of harnessing chromatin remodeling and epigenetic alteration in combating NAFLD. *KDM1A* might be considered as a leading therapeutic target in this regard.

## Materials and methods

### Animals

In this study, all animal experiments conformed to the Guide for the Care and Use of Laboratory Animals published by the National Academy of Sciences and the National Institutes of Health. The animal protocols and procedures were approved by the Animal Care and Use Committee of Renmin Hospital of Wuhan University and by the Institutional Animal Care and Use Committee at the Center for Animal Experiment, Wuhan University.

The hepatocyte-specific *Kdm1a*-KO (*Kdm1a* CKO) mice were generated using an AAV8 delivery system. To facilitate *Kdm1a* CKO mouse generation, specific primers (sg1: 5′-CTAAGTAACTGTGAACTCGG-3′; sg2: 5′- ACCAAGACCTGTTACAACCA-3′; sg3: 5′- AACCTCCAATGCCTGGCCAA-3′) were employed. Briefly, 5 × 1011 vg of AAV8 carrying specific guide RNA (sgRNA) sequences targeting the mouse *Kdm1a* gene was injected into C57BL/6J-Gt (ROSA)26Sortm1 mice. The sgRNA can work in conjunction with Cas9 nuclease to guide it to target specific genomic loci for DNA double-strand breaks. These mice were procured from the Model Animal Research Center of Nanjing University. The AAV8 vector was obtained from Vigenebio (Shandong, China). The tail vein route was selected for AAV8 injection.

To generate *Kdm1a*-overexpression (*Kdm1a*-OE) mice, we employed a Sleeping Beauty (SB) transposase system, following the protocol described in previous studies (20, 21). In brief, we introduced a liver-specific pT3 plasmid (pT3-alb-3×flag-m-*Kdm1a*), carrying the *Kdm1a* gene, into the mouse livers via tail vein injection. Each mouse received a dosage of 30 μg of the pT3 plasmid carrying the *Kdm1a* gene, along with 2 μg of the SB100X transposase plasmid. For the control group of mice, we only injected the SB100X transposase plasmid.

### Establishment of NAFLD model in mice

To establish a mouse model of NAFLD, male C57BL/6J mice were subjected to a high-fat high-cholesterol (HFHC) diet (fat, 42%; carbohydrates, 44%; protein, 14%; cholesterol, 0.2%; no. TP 26304, Trophic Diet, Nantong, China) for 16 weeks. Mice of the control group were fed with a normal chow diet (10.2% fat, 18.3% protein, and 71.5% carbohydrates; D12450B, Research Diets, New Brunswick, NJ).

### Human liver samples and animal liver samples

Human sample collection was approved by the human research ethics committee of Renmin Hospital of Wuhan University and Zhongnan Hospital of Wuhan University. The procedures adhered to the principles of the Declaration of Helsinki. Human liver tissue samples were obtained from individuals who underwent liver biopsy, liver surgery, or liver transplantation at Renmin Hospital of Wuhan University or Zhongnan Hospital of Wuhan University in Wuhan. Samples from patients with hepatic steatosis resulting from viral infection (such as hepatitis B or C virus), drug or toxin-induced injury, excessive alcohol consumption, or autoimmune hepatitis were excluded. The control samples of were collected from normal liver regions of individuals who underwent liver-related surgery and did not exhibit NAFLD or NASH. The NAFLD activity score was assessed independently by two pathologists in a blinded manner, utilizing the NASH-Clinical Research Network scoring system (22). Samples exhibiting an NAFLD activity score of 1–2 without ballooning or fibrosis were classified as simple steatosis, whereas those with an NAFLD activity score greater than four or an NAFLD activity score of 3–4 accompanied by the presence of fibrosis were classified as NASH. Nonsteatotic samples were characterized by an NAFLD activity score of 0. Informed consent was also obtained from all patients and volunteers involved in the study. The main characteristics of patients are shown in Supplemental Table S1. Cynomolgus monkey models of NAFLD or NASH were established by our group. For this study, liver biopsy tissues obtained from these primate models were utilized. All the protocols and procedures involving the monkeys had been approved by the Forestry Department of Hubei Province in China and strictly adhered to the laws and regulations of the People's Republic of China. In addition to the primate models, mouse liver tissues from models induced by a HFHC diet were also incorporated into our study.

### Hepatic lipid and serum biochemistry analyses

The hepatic lipid triglyceride (TG) contents were quantified by using commercial TG kits (A110-1-1, Nanjing Jiancheng Bioengineering Institute) according to the manufacturer’s directions. To assess the lipid content in the plasma and evaluate liver function, the serum levels of TGs, total cholesterol (TC), alanine aminotransferase (ALT), and aspartate aminotransferase (AST) were evaluated in the animals. For the analysis, an ADVIA 2400 Chemistry System analyzer (Siemens, Tarrytown, NY) was employed, following the guidelines provided by the manufacturer.

### Histological analysis and immunofluorescence staining

Tissues were fixed in a 10% neutral buffered formalin solution and subsequently embedded in paraffin for histological analysis. To visualize the lipid accumulation pattern and inflammatory status, paraffin liver sections were stained with H&E staining. For the detection of lipid droplet accumulation, frozen liver sections prepared in Tissue-Tek OCT compound were stained with Oil Red O (Sigma, St. Louis, MO). Liver fibrosis was assessed using picrosirius red staining (Hedebiotechnology, Beijing, China). The histological features of the stained tissues were observed and documented using a light microscope (ECLIPSE 80i, Nikon, Tokyo, Japan). In addition, for immunofluorescence staining, paraffin liver sections were labeled overnight with an anti-CD11b polyclonal antibody (BM3925, Boster, Wuhan, China) at a dilution of 1:800. Subsequently, the sections were incubated with a suitable secondary antibody conjugated with a fluorophore (1:200 dilution) for 1 h. The immunofluorescence images were captured using a Nikon fluorescence microscope (ECLIPSE 80i).

### Primary cell isolation and cell culture

To obtain murine primary hepatocytes and Kupffer cells, a two-step collagenase perfusion method was used. Primary hepatocytes and Kupffer cells were isolated from male C57BL/6J mice aged 6–8 weeks. Additionally, primary hepatic stellate cells (HSCs) were isolated from male C57BL/6J mice aged 16 weeks. Briefly, under anesthesia, the mice were perfused twice with liver perfusion medium (Thermo Fisher Scientific, Waltham, MA; catalog no.: 17701-038), followed by liver digestion medium (Thermo Fisher Scientific; catalog no.: 17701-034) through the liver portal vein. The liver tissue was excised, minced, and subsequently filtered through a 100 μm cell strainer. The resulting mixture was then subjected to centrifugation at 50 *g* for 5 min to separate the Kupffer cells (top aqueous phase) with the primary hepatocytes (the cell pellet). The aqueous phase was further subjected to purification using a 50% Percoll solution (17-0891-01; GE Healthcare Life Sciences) to obtain a highly purified population of Kupffer cells. In the case of HSCs, the cell suspension was centrifuged at 580 *g* and subsequently enriched using a 29% Nycodenz density gradient centrifugation method to achieve further enrichment. The isolated primary hepatocytes, Kupffer cells, and HSCs were cultured using DMEM supplemented with 10% fetal bovine serum and 1% penicillin-streptomycin. Huh7 cells and HEK293T (type culture collection of the Chinese Academy of Sciences) were maintained in DMEM supplemented with 10% fetal bovine serum (catalog no.: F05-001-B160216; Bio-One Biotechnology, Guangzhou) and 1% penicillin-streptomycin (catalog no.: 15140-122; Gibco). The cells were cultured in a controlled environment consisting of a 5% CO2 incubator maintained at 37°C.

### Generation of KO cell lines

Two sgRNAs were designed to target the human *KDM1A* genes and subsequently cloned into the lenti-CRISPRv2 plasmid. The constructed plasmid was used to generate the Cas9/sgRNA-producing lentivirus. The primer sequences used for constructing sgRNA-expressing plasmid were as follows (sg1:5′-GACTTCAAGACGACAGTTCTGG-3′ sg2:5′-TAAATAACTGTGAACTCGGTGG-3′). To generate lentivirus, the sgRNA-expressing plasmids, along with the packaging plasmids pMD2.G and psPAX2, were transfected into HEK293T cells. After 48 h of transfection, the lentivirus was obtained from the supernatant. The harvested cell culture supernatants underwent filtration using a 0.45-μm pore size membrane to remove cellular debris and contaminants. Subsequently, the filtered supernatants were subjected to ultracentrifugation for concentration, and the resulting concentrated viral particles were stored at −80°C until use. Then, the Huh7 cells were transduced with lentiviral supernatants, and three days posttransduction, puromycin was employed to select positive cells. Single cells were placed in wells of a 96-well plate separately to facilitate the growth of cell clones in the presence of puromycin for a duration of two weeks and then transferred to 24-well plates for another two weeks. Cell clones with the desired gene knockout were verified via Western blot (WB) analysis using the indicated antibodies and further confirmed by sequencing.

### Cellular BODIPY staining

Palmitic acid (P0500; Sigma–Aldrich, St. Louis, MO) and oleic acid (O-1008; Sigma–Aldrich) were used to induce stimulation and construct an in vitro cell model of NAFLD, as previously reported (23). For the control group, fatty acid–free BSA (0.5%; BAH66-0100; Equitech Bio, Kerrville, TX) was used. To visualize lipid droplets, cells were fixed using 4% paraformaldehyde and stained with BODIPY (D3922-10 mg; 1 μM in PBS; Thermo Fisher Scientific) for a duration of 3 min at room temperature. The cell nuclei were stained utilizing 4',6-diamidino-2-phenylindole staining. BODIPY images were acquired using a laser scanning confocal microscope (TCS SP8; Leica, Wetzlar, Germany).

### Cell viability assays

Cell viability was assessed using the Cell Counting Kit-8 assay kit, following the instructions provided by the manufacturer. In brief, huh7 hepatocytes were seeded at a density of 5,000 cells per well in 96-well plates. In brief, huh7 hepatocytes were seeded in 96-well plates at a density of 5,000 cells per well and treated with CC-90011 at concentrations of 1, 5, 10, and 20 μM or DMSO for 24 h. Following the treatment, cell counting was performed using Cell Counting Kit-8 (#B34304; Bimake; TX). The absorbance at 450 nm was measured for each well to estimate the number of viable cells.

### WB assay

For WB analysis, total protein was extracted from cultured cells and liver tissue samples using RIPA lysis buffer mixed with protease inhibitor cocktail tablets (Roche; 04693132001). The protein concentration was determined using a BCA Protein Assay Kit (Thermo Fisher Scientific; catalog no.: 23225). The protein samples were separated by SDS-PAGE, transferred to PVDF membranes (Millipore; catalog no.: IPVH00010), and blocked with 5% skim milk for 60 min at room temperature. Then, the membranes were incubated overnight at 4°C with primary antibodies, followed by incubation with the corresponding secondary antibodies. Imaging was performed using a ChemiDoc MP Imaging System (Bio-Rad, Hercules, CA). The antibodies used are detailed in Supplemental Table S2.

### RNA extraction and real-time PCR

Total RNA from cultured cells and tissues was isolated using TRIzol reagent (catalog no.: T9424; Sigma-Aldrich), and gene expression was quantified by real-time PCR using a LightCycler 480 (Roche Diagnostics, Inc, Basel, BS, Switzerland), following the manufacturer’s protocols. The relative expression levels of the target genes were normalized to the β-actin. The primers utilized for quantitative RT-PCR are listed in Supplemental Table S3.

### Dual-luciferase reporter assay

H293T cells were seeded in 24-well plates culture dishes. Afterward, H293T cells were cotransfected with pGL3-*KDM1A* promoter-luciferase plasmid, pRL-TK promoter luciferase reporter plasmid (Renilla luciferase), and PHAGE-Flag-*C/EBP-β*. The primers in the *KDM1A*-promoter construct used for the luciferase reporting assay are listed in Supplemental Table S4. Then, the cells were lysed and subjected to a luciferase activity assay using the Dual-Glo Luciferase Assay system (GloMax 20/20 Luminometer; Promega).

### Plasmid constructs

The PHAGE-Flag vector was used for the cloning of the full-length region of the human *KDM1A*, *HNF4α*, *C/EBP-α*, *C/EBP-β* gene. Expression plasmid containing mutant *KDM1A* gene was generated using established molecular biology methods. Human *C/EBP-β* knockdown plasmids were constructed by inserting shRNAtargeting *C/EBP-β* genes into the pLKO.1 lentiviral vector. The primers used for plasmid construction can be found in the Supplemental Table S4.

### Lentivirus and AAV production and infection

For the generation of recombinant lentiviruses, H293T cells were cotransfected with the lentiviral vector of interest and the helper plasmids psPAX2 and pMD2.G. Following a 48-h incubation period posttransfection, virus particles were filtered from the cell supernatant through a 0.45-μm filter (SLHV033RB; Millipore) and then used to infect 293T cells in the presence of 10 μg/ml polybrene. After puromycin screening, stably expressed cells were obtained and verified by WB. The AAV8 delivery system was employed to induce *Kdm1a* knockout in the liver of mice. For in vivo experiments, a dose of 5 × 10^11^ vg of AAV8 containing sgRNA sequences specifically targeting the mouse *Kdm1a* gene (obtained from Vigenebio, Shandong, China) was intravenously injected into the mice through the tail vein. The efficacy of gene knockout was assessed by performing WB analysis.

### RNA-sequencing and data analysis

The quality assessment of the extracted RNA samples was performed by using the RNA 6000 Nano kit (#5067-1511, Agilent, Santa Clara, CA), following the extraction procedure that utilized TRIzol reagent (#T9424, Sigma-Aldrich). For library preparation, the MGIEasy RNA Library Prep Kit (#1000006384, MGI Tech Co., Ltd, Shenzhen, China) was used. For the analysis of the obtained data, we used HISAT2 software (version 2.21) to align the clean reads against the Ensembl mouse genomes (mm10 or GRCm38). The resulting alignments were then converted to Binary Alignment Map format using SAMtools (version 1.4). To quantify the expression levels of the identified genes, we employed StringTie (version 1.3.3b) to calculate the fragments per kilobase per million and read counts. Subsequently, the obtained read counts were input into DESeq2 to determine differential gene expression and assess the associated statistical significance. Differentially expressed genes were identified as those with |log2 (fold change) |≥ log2(1.5) and an adjusted *P* value  < 0.05. In addition, gene set variation analysis analysis was performed by using the gene set variation analysis R package (v1.40.1) to evaluate the variation in pathway activity across different conditions. Statistical significance was determined for gene sets with *P* values  < 0.05.

### Assay for transposase-accessible chromatin using sequencing

Assay for transposase-accessible chromatin using sequencing (ATAC-seq) was conducted following established protocols using fresh liver tissues (19). The Bowtie2 alignment software was employed in this analysis. Prior to the alignment, the raw data obtained from sequencing was subjected to adapter trimming. The clean data was aligned to the mm10_UCSC reference genome using Bowtie2. The fragment size of the BAM files was calculated using the R package ATAC-seqQC. The uniquely mapped reads from the generated BAM files were used to call peaks using the macs2 software, with a significance threshold set at *P* value < 0.05. Subsequently, deeptools was used to compute signal distribution plots and heatmaps near genes. Differential peak analysis was performed using the R package DiffBind, followed by motif analysis using Multiple EM for Motif Elicitation-Chromatin Immunoprecipitation, and downstream gene prediction using MEME-Find Individual Motif Occurrences.

### Statistical analysis

Data analysis was conducted using SPSS v26, and the results are presented as means  ±  SEM. Differences between two groups with parametric data were evaluated using a Student’s *t* test, while a one-way ANOVA was employed for parametric data involving multiple comparisons. Post hoc tests, such as Bonferroni’s or Tamhane’s T2 (M), were performed to analyze significant findings, depending on the homogeneity of variance. In the case of skewed distribution, the Mann–Whitney U test and Kruskal–Wallis test were used for two and multiple-group comparisons, respectively. Statistical significance was defined as a *P* value  < 0.05.

## Results

### *KDM1A* expression is upregulated in NAFLD and correlates with the progression of NAFLD

**Fig. 1:**
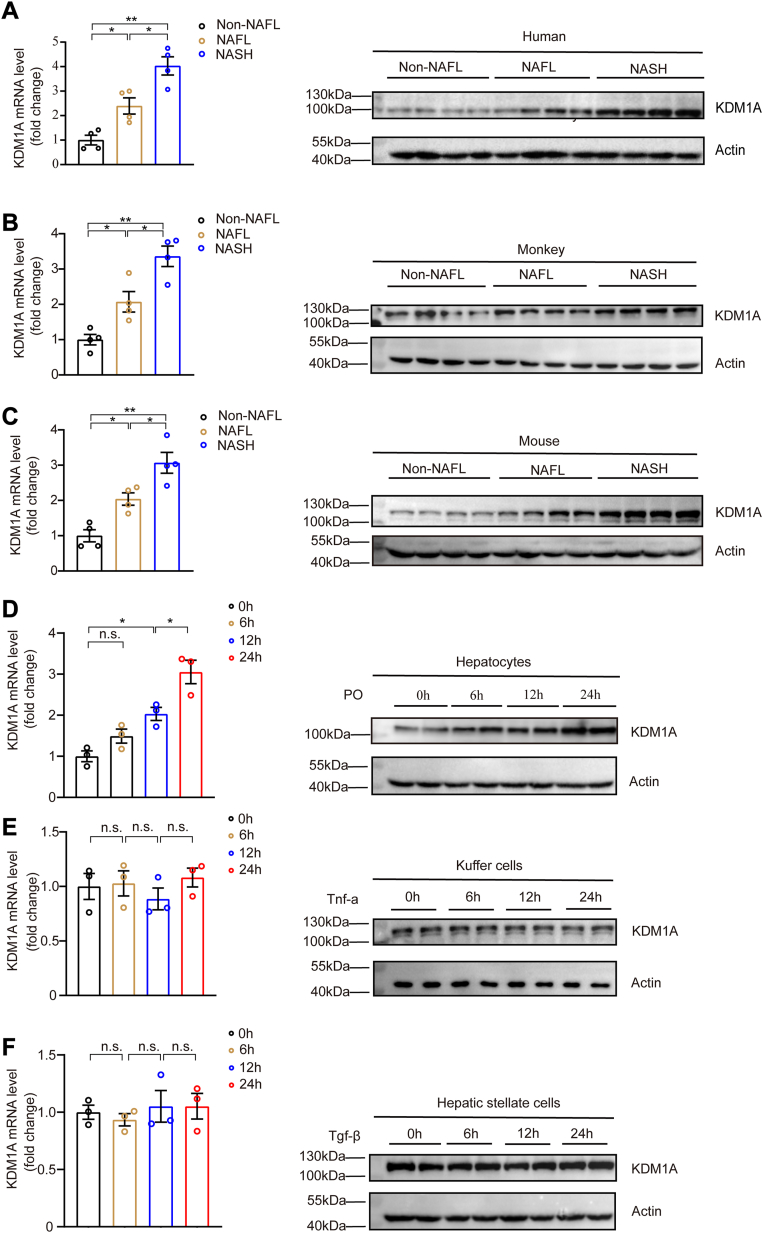
*KDM1A* is closely related to the progression of NAFLD. A: Quantitative PCR (qPCR) and Western blot analysis of *KDM1A* expression in the livers of non-NAFL, NAFL, and NASH individuals of humans (n = 4). B and C: The mRNA and protein level of *Kdm1a* in the liver tissues from non-NAFL, NAFL, and NASH mice and monkeys. D–F: Representative qPCR and Western blot analysis of *Kdm1a* expression in mouse primary hepatocytes, Kupffer cells, and hepatic stellate cells stimulated with 0.5 mM/1 mM palmitic acid/oil acid (PO), 10 ng/ml TNF-α, or 20 ng/ml transforming growth factor-β (TGF-β) in a time-dependent manner. n = 3 independent biological replicates. Values are presented as mean  ±  SD. ∗*P*  <  0.05, ∗∗*P*  <  0.01, n.s., not significant; one-way ANOVA statistics was applied for statistical analysis. NAFL, nonalcoholic fatty liver; NASH, nonalcoholic steatohepatitis.

To elucidate the association between NAFLD and *KDM1A*, we firstly determined the *KDM1A* level in patients and found significant upregulation of *KDM1A* expression in both mRNA and protein levels in the liver tissues of patients with NAFL/NASH (Fig. 1A). The same result was also observed in liver samples of in HFHC diet–induced monkeys and mice relative to the control groups and the upregulation level of *Kdm1a* increased with the progression of NAFLD (Fig. 1B, C). In primary hepatocytes, we also found that the *Kdm1a* mRNA and protein expression were markedly elevated in primary hepatocytes challenged with palmitic acid and oleic acid (PO) (Fig. 1D). Furthermore, the expression level of *Kdm1a* increased with the prolongation of PO stimulation time. Remarkably, the mRNA and protein levels of *Kdm1a* did not increase in Kupffer cells or HSCs (Fig. 1E, F). This result indicated that the observed elevation in *Kdm1a* expression was predominantly attributed to hepatocytes rather than Kupffer cells or HSCs. To investigate the possible mechanism by which palmitate activates *KDM1A*, we integrated the prediction of KDM1A promoter-binding transcription factors (TFs) (24, 25, 26). Among these TFs, *C/EBPα*, *C/EBPβ*, and *HNF4α* were NAFLD metabolism-related TFs (Supplemental Fig. S1A). We observed that *C/EBPβ* markedly increased the promotor activity of *KDM1A* and *KDM1A* mRNA levels (Supplemental Fig. S1B, C). In addition, *C/EBPβ* inhibition significantly decreased the mRNA level of *KDM1A* (Supplemental Fig. S1D, E). Collectively, these findings suggested that *C/EBPβ* was the upstream regulator of *KDM1A* and palmitate activated *KDM1A* through upregulating *C/EBPβ*.

### *KDM1A* promotes lipid accumulation and inflammation in hepatocytes

**Fig. 2:**
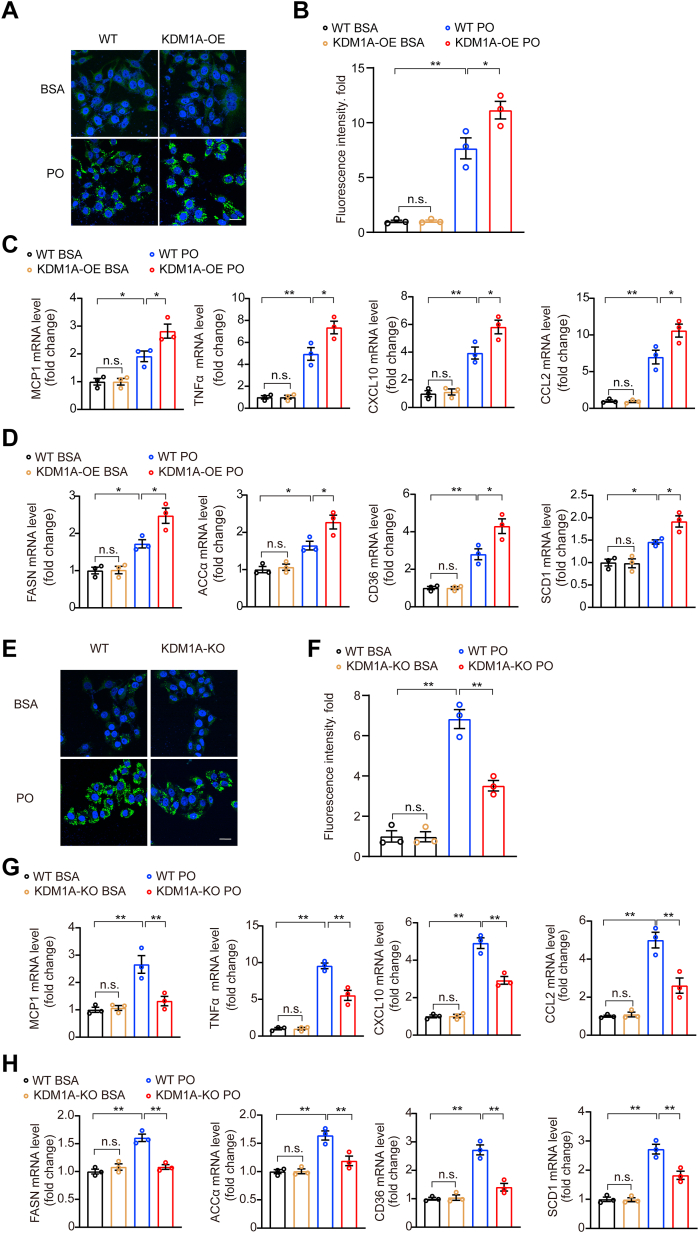
*KDM1A* accelerates hepatocyte lipid and inflammation disorder. A and B: Representative images (A) of BODIPY staining and quantification (B) of lipid droplets in cultured WT/(*KDM1A*-OE) Huh7 cells challenged with BSA or PO for 12 h under the indicated conditions. n  =  3 independent biological replicates. Scale bar 25 μm. C: Relative mRNA levels of genes associated with inflammation in cultured WT/*KDM1A*-overexpression (*KDM1A*-OE) Huh7 cells stimulated with BSA or PO at the indicated concentrations for 16 h. The data shown are representative of three independent experiments. D: Relative mRNA levels of genes associated with fatty acid uptake and fatty acid synthesis in cultured WT/*KDM1A*-OE Huh7 cells stimulated with BSA or PO at the indicated concentrations for 16 h. The data shown are representative of three independent experiments. E and F: Representative images (E) of BODIPY staining and quantification (F) of lipid droplets in cultured WT/(*KDM1A*-KO) Huh7 cells challenged with BSA or PO for 12 h under the indicated conditions. n  =  3 independent biological replicates. Scale bar 25 μm. G: Relative mRNA levels of genes associated with inflammation in cultured WT/*KDM1A*-KO (*KDM1A*-KO) Huh7 cells stimulated with BSA or PO at the indicated concentrations for 16 h. The data shown are representative of three independent experiments. H: Relative mRNA levels of genes associated with fatty acid uptake and fatty acid synthesis in cultured WT/(*KDM1A*-KO) Huh7 cells stimulated with BSA or PO at the indicated concentrations for 16 h. The data shown are representative of three independent experiments. Values are presented as mean ± SD. ∗*P* < 0.05, ∗∗*P* < 0.01, n.s., not significant; one-way ANOVA statistics was applied for statistical analysis. PO, palmitic acid and oleic acid.

Given the close correlation between *KDM1A* and the progression of NAFLD, we further examined the functions of *KDM1A* in vitro and found a significant increase in lipid accumulation induced by PO in hepatocytes overexpressing *KDM1A* (Fig. 2A, B). Consistent with these findings, we observed a significant upregulation of *MCP1*, *TNFα*, *CXCL10*, *CCL2*, *FASN*, *ACCα*, *CD36*, and *SCD1* in hepatocytes overexpressing *KDM1A* (Fig. 2C, D). Conversely, the depletion of *KDM1A* reduced lipid droplet accumulation within hepatocytes following PO stimulation (Fig. 2E, F). And *KDM1A* depletion reduced mRNA expression levels for *MCP1*, *TNFα*, *CXCL10*, *CCL2*, *FASN*, *ACCα*, *CD36*, and *SCD1* (Fig. 2G, H). Taken together, these findings suggest that *KDM1A* can promote lipid accumulation and inflammation in hepatocytes.

### Hepatocyte-specific *Kdm1a* ablation alleviates HFHC diet–induced NAFLD in mice

**Fig. 3:**
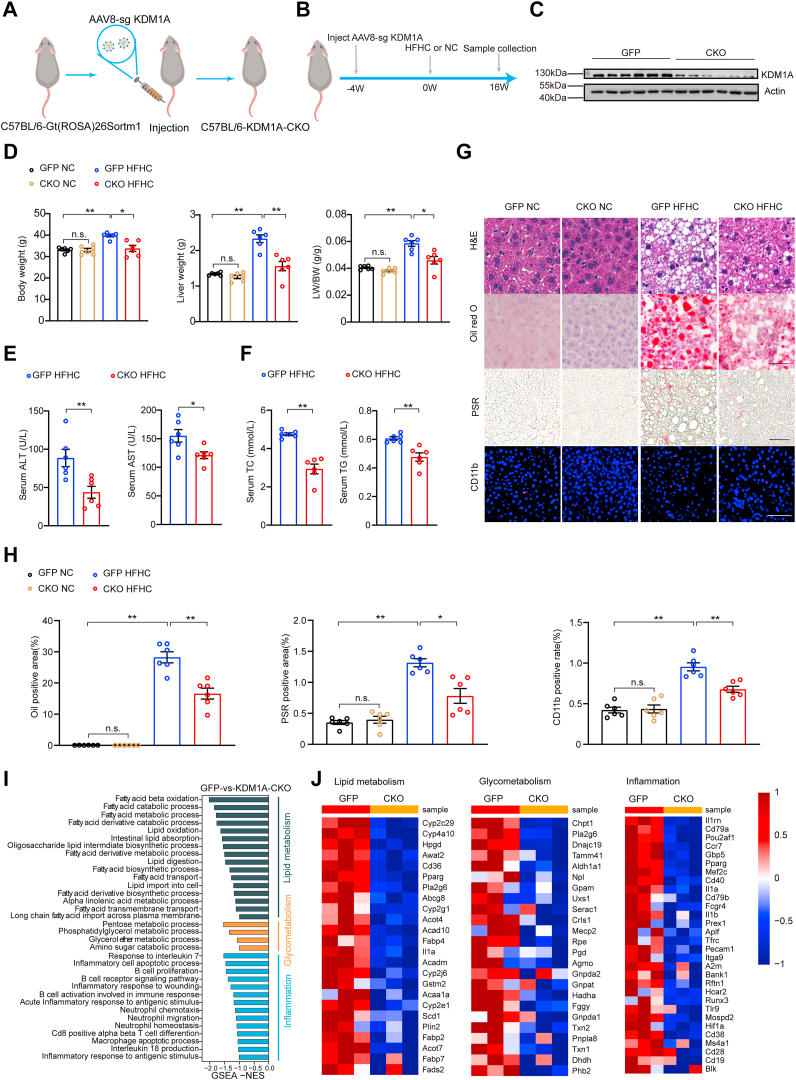
Deletion of *Kdm1a* improves HFHC-induced NAFLD in mice. A: Schematic diagram for generating hepatocyte-specific *Kdm1a*-KO (*Kdm1a* CKO) mice. B: The strategy of the experimental procedure for the HFHC-induced NAFLD model and evaluating the *Kdm1a* function in vivo. C: Representative Western blot images of the *Kdm1a* protein expression at the 16th week. n  =  6 mice per group. D: Body weight, liver weight, and the ratio of liver weight to body weight (LW/BW) of HFHC-fed mice in the indicated groups. n  =  6 mice per group. E: Serum alanine transaminase (ALT) and aspartate transaminase (AST) activity of HFHC-fed mice were shown in the indicated groups. n  =  6 mice per group. F: Serum total cholesterol (TC) and triglyceride (TG) concentrations of HFHC-fed mice are shown in the indicated groups. n  =  6 mice per group. G and H: Representative images (G) of the indicated mouse liver sections stained with H&E, oil red O, picrosirius red (PSR), and immunofluorescence for CD11b-positive cells. n  =  6 mice per group. Scale bar, 50 μm. Quantitative analysis of oil red O, PSR, and CD11b data is shown in (H). n  =  6 mice per group. I: Enrichment of Gene set variation analysis (GSVA) pathways related to lipid metabolism, glycometabolism, and inflammation. n  =  3 mice per group. J: Heatmap of gene expression profiles involved in lipid metabolism, glycometabolism, and inflammation. n  =  3 mice per group. Values are presented as mean  ±  SD. ∗*P*  <  0.05, ∗∗*P*  <  0.01, n.s., not significant; one-way ANOVA statistics was applied in (D, H). Student's *t* test was applied in (E, F). HFHC, high-fat high-cholesterol; NAFLD, nonalcoholic fatty liver disease; PSR, picrosirius red.

Based on the vital role of *Kdm1a* in NAFLD models in vitro, we generated *Kdm1a* CKO mice to explore the function of *Kdm1a* in vivo (Fig. 3A). We subjected the *Kdm1a* CKO mice together with the controls to either a normal chow or HFHC diet for a duration of 16 weeks to make controls or induce NAFLD (Fig. 3B). Moreover, we validated the successful knockout of *Kdm1a* specifically in the liver (Fig. 3C). After 16 weeks of HFHC feeding, the *Kdm1a*-deficient mice exhibited lower body weight, liver weight, and liver weight/body weight ratios than the control mice (Fig. 3D). Meanwhile, the levels of liver injury markers, including ALT and aspartate AST, the levels of liver TG as well as serum TC and TG concentrations, were markedly reduced in the *Kdm1a* CKO group (Fig. 3E, F, Supplemental Fig. S2A). Moreover, *Kdm1a* deficiency led to a substantial reduction in the accumulation of hepatic lipid droplets and significantly mitigated inflammatory cell infiltration and fibrosis (Fig. 3G, H). Upon performing a comprehensive global transcriptome analysis of HFHC-fed mouse livers, we discovered that pathways related to lipid metabolism, glycometabolism, and inflammation were repressed in *Kdm1a* CKO mice (Fig. 3I). Correspondingly, the expression of key genes implicated in these pathways associated with lipid metabolism, glycometabolism, and inflammation was also suppressed in the *Kdm1a* CKO group (Fig. 3J). In *Kdm1a* CKO group, we observed an improvement in lipid metabolism, primarily attributed to the downregulation of genes related to lipid biosynthesis and uptake (Supplemental Fig. S3A, B). However, the expression of genes related to lipid degradation remained unchanged (Supplemental Fig. S3C). In addition, our research findings also indicated that *Kdm1a* CKO mice showed a significant decrease in the expression levels of genes involved in gluconeogenesis compared to the control group (Supplemental Fig. S4A).

### Overexpression of *Kdm1a* exacerbates HFHC diet-induced NAFLD in mice

**Fig. 4:**
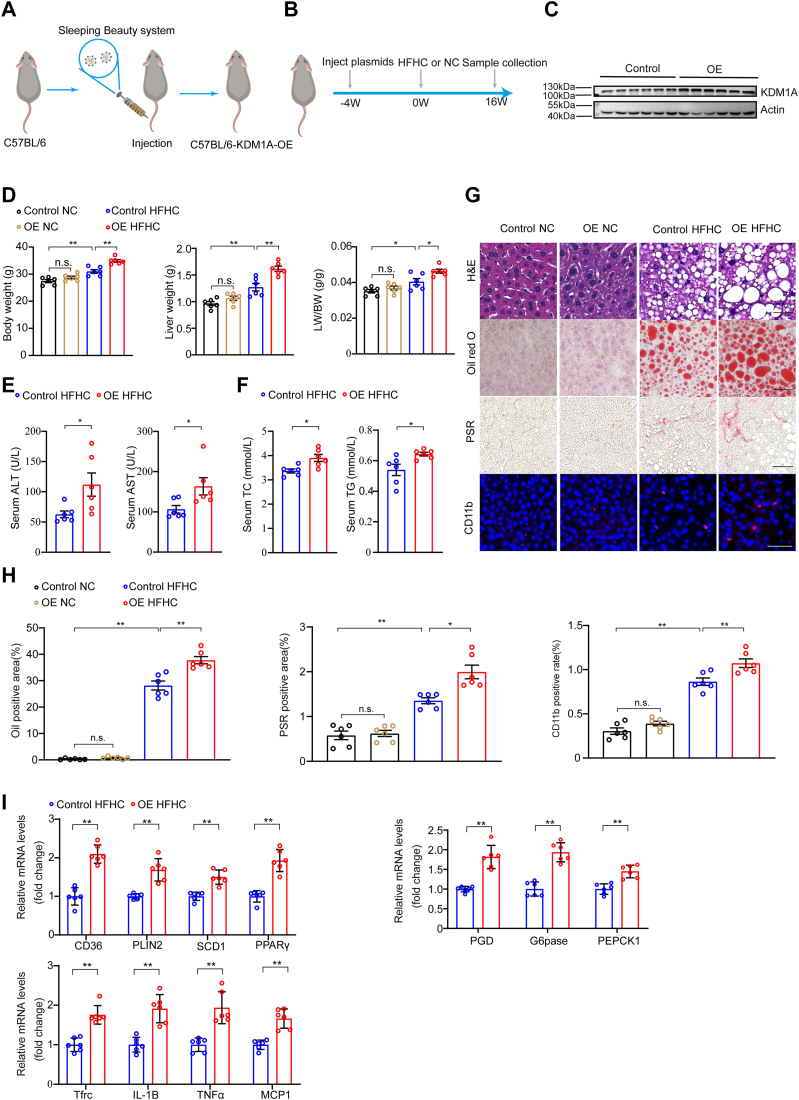
Overexpression of *Kdm1a* aggravates HFHC-induced NAFLD in mice. A: Experimental schematic for generating *Kdm1a*-OE mice. B: Schematic showing HFHC-induced NAFLD and evaluation of the function of *Kdm1a*. C: Western blot analysis of the *Kdm1a* protein expression at the 20th week. n  =  6 mice per group. D: Body weight, liver weight, and LW/BW of HFHC-fed mice in the indicated groups. n  =  6 mice per group. E: Serum ALT and AST activity of HFHC-fed mice are shown in the indicated groups. n  =  6 mice per group. F: Serum TC and TG levels of HFHC-fed mice are shown in the indicated groups. n  =  6 mice per group. G and H: Representative images (G) of H&E, oil red O, PSR-stained liver sections, and Immunofluorescence for CD11b-positive cells. n  =  6 mice per group. Scale bar, 50 μm. Quantitative analysis of oil red O, PSR, and CD11b data is shown in (H). n  =  6 mice per group. I: Relative mRNA levels of genes related to inflammatory response, glycometabolism, and lipid metabolism in the livers of HFHC diet–fed mice with or without *Kdm1a*-OE. n = 6 mice per group. Values are presented as mean  ±  SD. ∗*P*  <  0.05, ∗∗*P*  <  0.01, n.s., not significant; one-way ANOVA statistics was applied in (D, H). Student's *t* test and Mann–Whitney U test were applied in (E, F, I). ALT, alanine aminotransferase; AST, aspartate aminotransferase; HFHC, high-fat high-cholesterol; *Kdm1a*-OE, *Kdm1a*-overexpression; NAFLD, nonalcoholic fatty liver disease; PSR, picrosirius red.

We next generated the *Kdm1a*-OE mice to further elucidate the crucial role of *Kdm1a* in NAFLD (Fig. 4A). The mice were subjected to a HFHC diet for a duration of 16 weeks to establish the NAFLD model (Fig. 4B). The successful overexpression of *Kdm1a* in the mouse liver was also confirmed (Fig. 4C). The *Kdm1a*-OE mice exhibited significantly higher liver weight, body weight, and liver weight-to-body weight ratio than the control group (Fig. 4D). Furthermore, there was a significant increase in AST, ALT, TC, and TG levels in *Kdm1a*-OE mice (Fig. 4E, F, Supplemental Fig. S2B). The overexpression of *Kdm1a* exacerbated hepatic steatosis, inflammation, and fibrosis induced by a HFHC diet (Fig. 4G, H). Consistent with these phenotypic alterations, *Kdm1a* overexpression resulted in a significant upregulation of key genes involved in lipid metabolism, glycometabolism, and inflammatory response (Fig. 4I, Supplemental Fig. S4B). These findings demonstrated that *Kdm1a* exacerbated hepatic steatosis, inflammation, and glucose disturbances in mice.

### *Kdm1a* deficiency downregulates the genes associated with NAFLD by decreasing chromatin accessibility

**Fig. 5:**
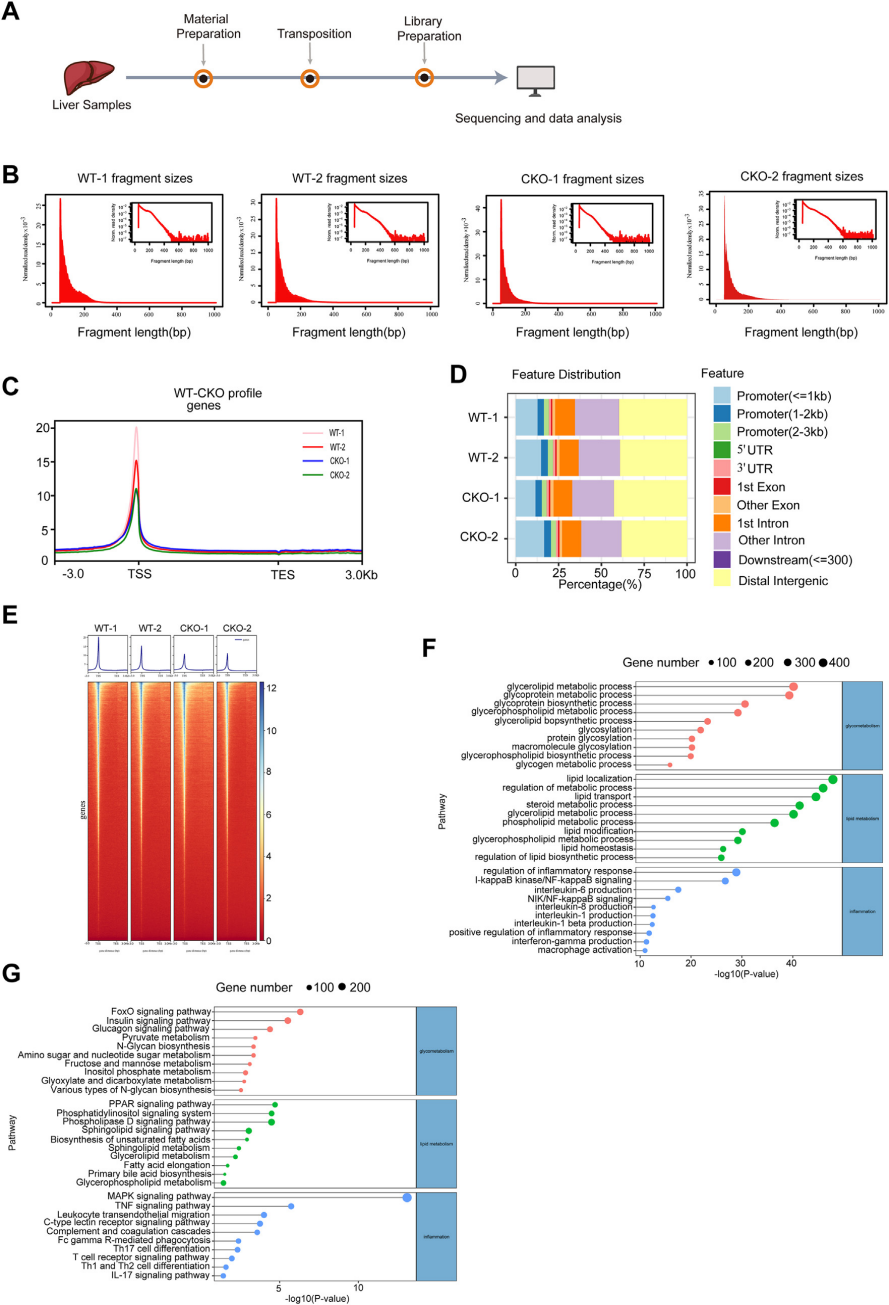
Chromatin accessibility landscape of WT and CKO groups. A: Schematic overview of ATAC-seq procedure. B: The insert size distribution of ATAC-seq profiles in WT and *Kdm1a*-CKO mice. C: Chromatin accessibility around the transcription start site (TSS) in WT and *Kdm1a*-CKO mice. D: Distribution of peaks in each sample. E: Average plots (top) and heatmaps (bottom) of differentially accessible regions (DARs) in WT and *Kdm1a*-CKO groups. Average plot showing global chromatin accessibility changes across the center and surrounding regions (±3 kb). The heatmap represents the average normalized ATAC-seq counts for DARs. Each row represents one DAR. F: Functional enrichment of gene ontology (GO) terms for genes associated with peaks in the WT versus *Kdm1a* CKO groups. G: The Kyoto Encyclopedia of Genes and Genomes (KEGG) annotation for the genes associated with peaks in the WT versus *Kdm1a* CKO groups. ATAC-seq, assay for transposase-accessible chromatin using sequencing; *Kdm1a*-CKO, hepatocyte-specific *Kdm1a*-KO.

Histone demethylase *KDM1A* has been reported to affect chromosome accessibility (27, 28). Therefore, to explore the potential mechanism underlying the critical roles of *KDM1A* in NAFLD, ATAC-seq was performed on liver samples of WT and *Kdm1a* CKO mice (Fig. 5A). The chromatin accessibility fragments of WT and *Kdm1a* CKO groups showed mainly a mononucleosome peak (Fig. 5B). The accessibility of the transcription start site was significantly enriched in both WT and *Kdm1a* CKO groups (Fig. 5C). These results showed that high-resolution chromatin accessibility and good quality of ATAC-seq were obtained from liver tissue samples of WT and *Kdm1a* CKO mice. The proportion of promoter regions was less than 25%, while the proportion of intron and distal intergenic was more than 50% in both WT and *Kdm1a* CKO groups (Fig. 5D). Moreover, the chromatin accessibility of the *Kdm1a* CKO group was decreased compared to the WT group at the transcription start site (Fig. 5E). Genes around differentially accessible regions were closely associated with glycometabolism, lipid metabolism, and inflammation in both WT and *Kdm1a* CKO groups (Fig. 5F, G).

**Fig. 6:**
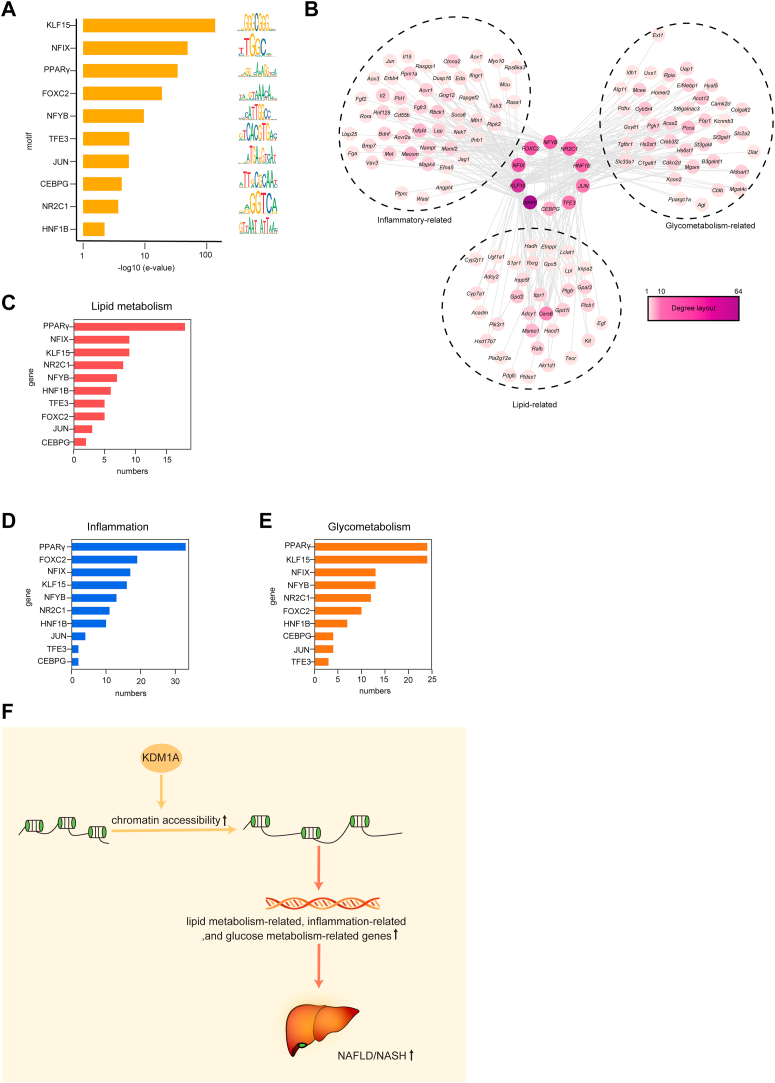
*Kdm1a* deficiency downregulates the genes related to NAFLD. A: Motif-enrichment analysis of differentially accessible sites in the WT versus *Kdm1a* CKO groups and motifs of the top 10 transcription factors are shown. B: The target genes of transcription factors, these target genes associated with inflammation, lipid metabolism, and glucose metabolism. C–E: Transcription factors were ranked based on the number of target genes associated with (C) lipid metabolism, (D) inflammation, and (E) glycometabolism. F: Schematic showing the mechanism that *Kdm1a* contributes to the progression of NAFLD by increasing chromatin accessibility. *Kdm1a*-CKO, hepatocyte-specific *Kdm1a*-KO; NAFLD, nonalcoholic fatty liver disease.

The 10 most enriched TF motifs in the *Kdm1a* CKO and WT groups are shown (Fig. 6A). The 10 TF motifs were associated with inflammation, lipid metabolism, and glucose metabolism. We next confirmed the target genes related to inflammation, lipid metabolism, and glucose metabolism for the top 10 identified TFs (Fig. 6B). Compared with the WT group, these target genes were around the low openness regions in the *Kdm1a* CKO group. These ATAC-seq results were consistent with the RNA-sequencing results in the Fig. 3J. Remarkably, the TF *Pparγ* had the highest number of target genes located in low openness regions, whether in terms of lipid metabolism, inflammation, or glucose metabolism (Fig. 6C–E). In addition, we observed a higher correlation between *Pparγ* and ATAC-seq than the TF *Klf15* (Supplemental Fig. S5A, B). Overall, our findings indicated that *Kdm1a* participates in the development of NAFLD by increasing chromosome accessibility (Fig. 6F).

### The effect of *KDM1A* on NAFLD depends on its enzyme activity

**Fig. 7:**
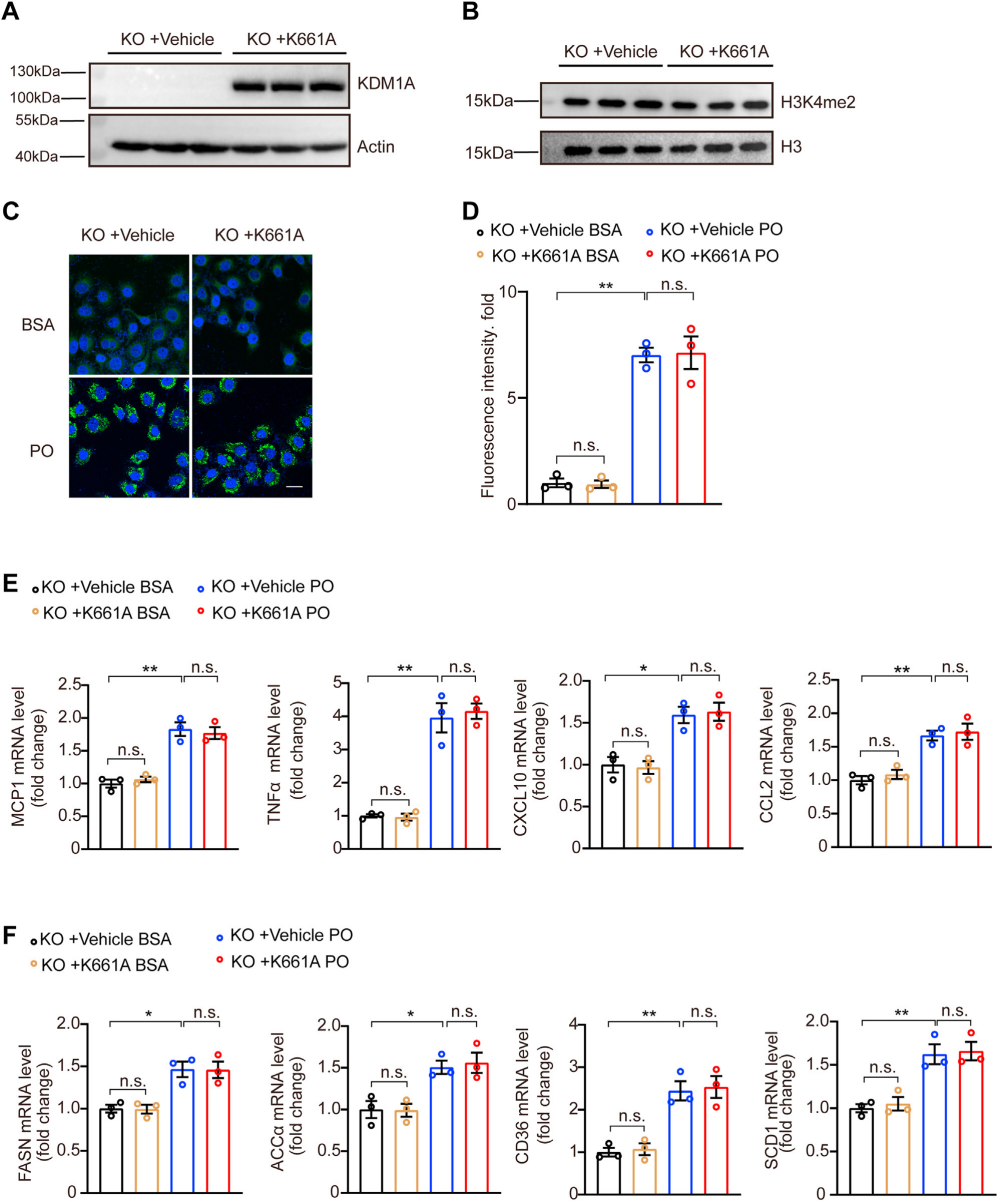
The enzyme activity of *KDM1A* is necessary for affecting NAFLD. A: Western blot image showing *KDM1A* protein expression levels in *KDM1A*-KO Hun7 hepatocytes transfected with empty vector or K661A plasmids. B: Histone demethylase activity was analyzed by anti-H3K4me2. The data shown are representative of three independent experiments. C and D: Representative images (C) of BODIPY staining and quantification (D) of fluorescence in *KDM1A*-KO hun7 hepatocytes transfected with empty vector or K661A plasmids challenged with BSA or PO for 12 h under the indicated conditions. n  =  3 independent biological replicates. Scale bar 25 μm. E: Relative mRNA levels of genes associated with inflammatory response in cultured *KDM1A*-KO hun7 hepatocytes transfected with empty vector or K661A plasmids stimulated with BSA or PO at the indicated concentrations for 16 h. The data shown are representative of three independent experiments. F: Relative mRNA levels of genes associated with lipid metabolism in cultured *KDM1A*-KO hun7 hepatocytes transfected with empty vector or K661A plasmids stimulated with BSA or PO at the indicated concentrations for 16 h. The data shown are representative of three independent experiments. Values are presented as mean  ±  SD. ∗*P*  <  0.05, ∗∗*P*  <  0.01, n.s., not significant; one-way ANOVA statistics was applied for analysis. NAFLD, nonalcoholic fatty liver disease; PO, palmitic acid and oleic acid.

Given that the demethylase activity of *KDM1A* is vital for its participating in various diseases such as lung adenocarcinoma and acute myeloid leukemia, we sought to investigate whether the enzymatic activity of *KDM1A* is indispensable for its pivotal role in the development of NAFLD (29, 30, 31, 32). Previous studies have reported that the mutant form of *KDM1A* (K661A) lacks the ability to demethylate histone H3 lysine 4 (33). We also confirmed that the K661A indeed lost catalytic activity (Fig. 7A, B). In *KDM1*A-KO cells, the re-expression of the K661A did not significantly restore the lipid accumulation induced by PO in hepatocytes compared to *KDM1A*-KO hepatocyte controls (Fig. 7C, D). Moreover, we observed no significant changes in the mRNA expression levels of *MCP1*, *TNFα*, *CXCL10*, *CCL2*, *FASN*, *ACCα*, *CD36*, and *SCD1* in *KDM1A*-knockout hepatocytes transfected with the mutant *KDM1A* (Fig. 7E, F).

### The *KDM1A* inhibitor can alleviate lipid accumulation and inflammation in hepatocytes

**Fig. 8:**
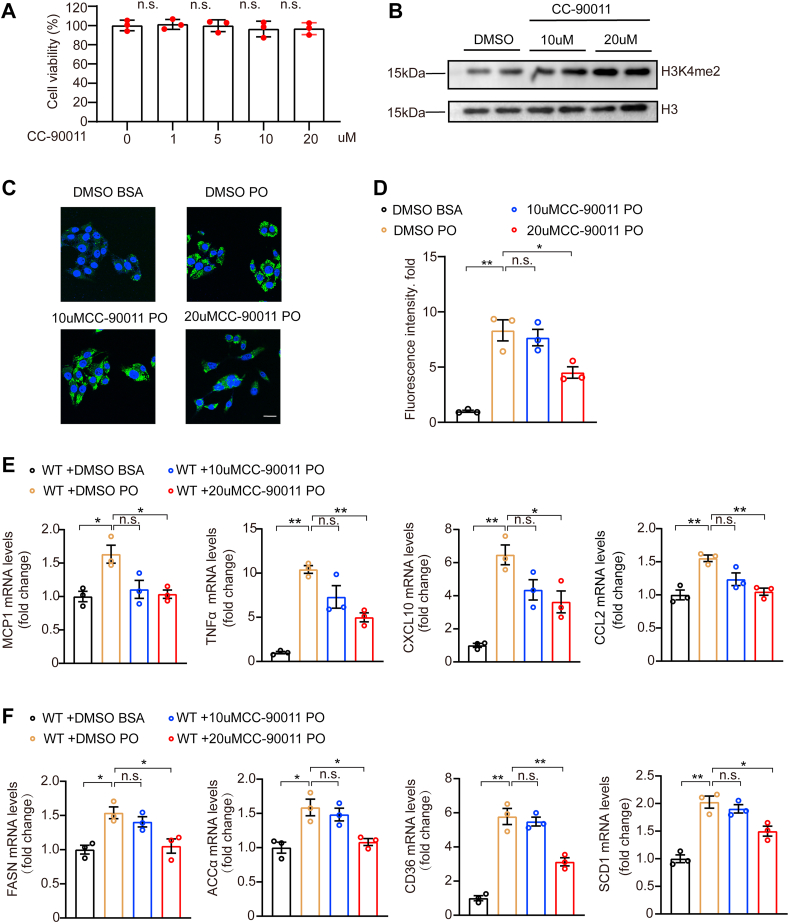
The *KDM1A* inhibitor alleviates lipid accumulation and inflammation. A: Relative cell viability of WT hun7 hepatocyte after treatment with different concentrations of CC-90011. The data shown are representative of three independent experiments. n.s., no significant difference compared to the 0 μM group. B: Western blot images showing the effect of CC-90011. The data shown are representative of three independent experiments. C and D: Representative images (C) of BODIPY staining and quantification (D) of lipid droplets in WT hun7 hepatocyte treated with DMSO+BSA, DMSO+PO, CC-90011 (10 or 20 μM) +PO for 12 h. n  =  3 independent biological replicates. Scale bar 25 μm. E: Relative mRNA levels of genes associated with inflammation in WT hun7 hepatocytes treated with DMSO+BSA, DMSO+PO, CC-90011 (10 or 20 μM) +PO for 16 h. The data shown are representative of three independent experiments. F: Relative mRNA levels of genes associated with fatty acid uptake and fatty acid synthesis in WT hun7 hepatocytes treated with DMSO+BSA, DMSO+PO, CC-90011 (10 or 20 μM) +PO for 16 h. The data shown are representative of three independent experiments. Values are presented as mean  ±  SD. ∗*P* < 0.05, ∗∗*P* < 0.01, n.s., not significant; one-way ANOVA statistics was applied for analysis. PO, palmitic acid and oleic acid.

We further applied CC-90011, a potent *KDM1A* enzyme inhibitor, in the cellular model of NAFLD to confirm the enzyme activity of *KDM1A* is a key factor promoting NAFLD. CC-90011 did not affect cell viability at the highest concentration (20 μM) used in our study (Fig. 8A). CC-90011 indeed inhibited the enzyme activity of *KDM1A* (Fig. 8B). We found that CC-90011 treatment dose dependently inhibited the increase in lipid accumulation in hepatocytes (Fig. 8C, D). Meanwhile, treatment with CC-90011 markedly reduced the mRNA expression levels of *MCP1*, *TNFα*, *CXCL10*, *CCL2*, *FASN*, *ACCα*, *CD36*, and *SCD1* in hepatocytes stimulated with PO (Fig. 8E, F). Collectively, these findings underscored the crucial role of *KDM1A*’s enzymatic activity in promoting the development of NAFLD.

## Discussion

NAFLD has become one of the most prevalent chronic liver conditions, affecting more than a quarter of the global population (2, 34). No specific drugs have been approved by the Food and Drug Administration, mainly due to the limited understanding of the underlying molecular mechanisms of NAFLD. We here discovered that histone demethylase *KDM1A* exacerbates hepatic steatosis and inflammation in NAFLD via increasing chromatin accessibility, indicating harnessing chromatin remodeling and epigenetic alteration may be a potential way to combat NAFLD. *KDM1A* itself might be considered as a leading therapeutic target against NAFLD.

Although the pathogenesis of NAFLD has not been fully elucidated, a large number of studies have shown that inflammation, dyslipidemia, and intestinal dysbiosis play a crucial role in the development and progression of NAFLD (35, 36, 37). Furthermore, several significant small molecules regulating inflammation, dyslipidemia, and gut microbiota in NAFLD have been developed as potential drug treatment targets (38, 39, 40). However, the outcomes of the corresponding clinical trials have so far been unsuccessful due to various reasons (40). Accumulating evidence indicates that chromatin remodeling and epigenetic modifications play important regulatory roles in the development of many diseases including NAFLD (9, 11). Our study revealed that increased expression of *KDM1A* can potentiate hepatic steatosis and inflammation by increasing chromatin accessibility in the genomic regions related to the pathogenesis of NAFLD, further indicating that dynamic changes in chromosomal structure under metabolic stress can lead to the development of NAFLD. Therefore, chromatin remodeling and epigenetic modifications are also crucial pathogenic factors in the development of NAFLD. *KDM1A* can be considered as a potential therapeutic target for NAFLD.

In this study, we found that *Kdm1a* deficiency in the context of NAFLD improved hepatic steatosis by primarily inhibiting the expression of lipogenesis and fatty acid uptake genes, such as *Scd1*, *Cd36*, *Plin2*, and *Fabp1*. Furthermore, *Kdm1a* deficiency in the context of NAFLD also suppressed the expression of several inflammatory genes, such as *Tnfα* and interleukin-1β, to ameliorate inflammation. In addition to improving lipid metabolism and inflammation, KDM1A deficiency also led to downregulation of gluconeogenic genes such as *G6pase* and *Fbpase*, suggesting that *Kdm1a* deficiency was also beneficial for glucose homeostasis. Mechanistically, we found that *Kdm1a* deficiency led to an improvement in hepatic steatosis and inflammation by decreasing the chromatin openness in regions where genes associated with inflammation, lipid metabolism, and glucose metabolism are located. *KDM1A* belongs to the histone demethylase family. Several members of histone demethylases have been reported to mediate metabolic crosstalk between tissues and organs, thereby playing a pivotal role in the development and progression of NAFLD (13). For instance, plant homeodomain finger 2 protects the liver from the pathogenesis of NAFLD by promoting H3K9me2 demethylation at promoters of carbohydrate responsive element binding protein–regulated genes (41). The histone demethylase *KDM7A* has been found to promote liver steatosis by upregulating the expression of diacylglycerol acyltransferase 2 (42). As the first identified lysine demethylase, the function of *KDM1A* in NAFLD has never been reported, although the potential role of *KDM1A* has been explored in various fields. It has been reported that *KDM1A* can be involved in prostate cancer by increasing chromatin accessibility of the *FOXA1* promoter (28). In addition, a study indicated that inhibition of *KDM1A* caused a global elevation of chromatin accessibility in acute myeloid leukemia cells (43). To determine whether and how *KDM1A* participates in the pathogenesis of NAFLD, we undertook systemic in vitro and in vivo functional experiments and an analysis employing ATAC-seq exploration. We found that depletion of *KDM1A* decreased the chromatin openness in genomic regions related to genes associated with inflammation, lipid metabolism, and glucose metabolism. Consequently, *KDM1A* deficiency ameliorated inflammation and lipid disorders in the liver. This mechanism is different from what we learn from other histone demethylases mentioned above, particularly in the pathogenesis of NAFLD.

It has been reported that *KDM1A* may have additional biological functions except for the demethylase activity of *KDM1A* (44). For instance, *KDM1A* facilitates the degradation of *FBXW7* via both proteasomal and lysosomal pathways independent of its demethylase activity (45). Therefore, we determined to explore whether the enzyme activity of *KDM1A* is crucial for NAFLD. In the present study, we observed that the supplementation of *KDM1A*-mutant (K661A) in *KDM1A*-KO hepatocytes showed no significant impact on lipid accumulation and inflammatory response. In line with these results, the administration of *KDM1A* inhibitor (CC-90011) demonstrated a notable protective effect against NAFLD. These compelling findings provide evidence supporting the notion that the influence of *KDM1A* on NAFLD is dependent on its enzymatic activity, highlighting its potential as a promising therapeutic target for the treatment of NAFLD.

A notable advantage of considering *KDM1A* as a therapeutic target for NAFLD is the availability of several targeted inhibitors that have been developed against it. Some of these inhibitors, including CC-90011 and TCP, have even progressed to clinical trials and have exhibited encouraging therapeutic effects in cancers (46, 47). Therefore, considering the good in vivo activity and low toxicity of *KDM1A* inhibitors, its application in the clinical treatment of NAFLD is promising. It is possible to screen the most effective *KDM1A* inhibitor in animal models like mice and then move on to clinical trials of NAFLD. Moreover, novel compounds targeting *KDM1A* have been emerging in recent years. In order to optimize the clinical efficacy of *KDM1A* inhibitors, we believe that at least a few key aspects should be emphasized, such as extensive safety assessments, the optimal dosage regimen, and a comprehensive evaluation of the pharmacokinetic characteristics of *KDM1A* inhibitors. Collectively, these may drive the imminent clinical utilization of safe and efficacious *KDM1A* inhibitors in the management of NAFLD in future.

To further evaluate whether *KDM1A* could be developed as a drug target against NAFLD, it is indispensable to learn the global function of KDM1A beyond its role in liver. In this regard, constructing and applying universal *Kdm1a* transgenic or KO mouse models would be necessary. However, previous studies have reported that global *Kdm1a* deletion is lethal during embryonic development (48, 49). Consequently, our future research endeavors will focus on the global *Kdm1a* transgenic and inducible KO models.

In conclusion, our study reveals that *KDM1A* aggravates liver steatosis and inflammation through increasing the accessibility of chromatin, demonstrating that harnessing chromatin remodeling and epigenetic modifications is crucial for managing NAFLD.

## Data availability

All data are contained within the manuscript and Supplementary data.

## Supplemental data

This article contains supplemental data.

## Conflict of interest

The authors declare that they have no conflicts of interest with the contents of this article.
