# Supplementary material for: Histone demethylase KDM1A promotes hepatic steatosis and inflammation by increasing chromatin accessibility in NAFLD
Source: J Lipid Res. 2024 Jan 29;65(3):100513. doi: 10.1016/j.jlr.2024.100513 (PMC10907224; doi:10.1016/j.jlr.2024.100513)
Supplement: Supplemental Data [file mmc1.docx]

# Histone demethylase KDM1A promotes hepatic steatosis and inflammation by increasing chromatin accessibility in NAFLD

Zifeng Yang^1,2,‡^, Siyao Zhang^3,‡^, Xiang Liu^3^, Rui Shu^2,6^, Wei Shi^2,6^,Weiyi Qu^2,4^, Dianyu Liu^3^, Zhiwei Cai^2^, Ye Wang^3^, Xu Cheng^3^, Yemao Liu^5^, Xiao-Jing Zhang^2,6^, Lan Bai^3,^*, Hongliang Li^1,2,3,6,^*, Zhi-Gang She^1,2,^*

^1^ Department of Cardiology, Renmin Hospital of Wuhan University, Wuhan, China

^2^ Institute of Model Animal, Wuhan University, Wuhan, China

^3^ Gannan Innovation and Translational Medicine Research Institute, State Key Laboratory of New Targets Discovery and Drug Development for Major Diseases, Gannan Medical University, Ganzhou, China

^4^Department of Cardiology, Zhongnan Hospital of Wuhan University; Wuhan, China.

^5^Department of Cardiology, Huanggang Central Hospital, Huanggang, China

^6^ School of Basic Medical Sciences, Wuhan University, Wuhan, China

*For correspondence: zgshe@whu.edu.cn (Zhi-Gang She), lihl@whu.edu.cn (Hongliang Li), bailan@gmu.edu.cn (Lan Bai).

^‡^These authors contributed equally.

**Supplemental Table S1. Main characteristics of patients**

| Parameters | Non-NAFL | NAFL | NASH |
| --- | --- | --- | --- |
| N=4 per group |  |  |  |
| Sex (male/female) | 2/2 | 2/2 | 1/3 |
| Age (years) | 43.0±11.6 | 41.0±8.8 | 31.5±4.8 |
| Weight (kg) | 78.5±4.5 | 95.0±15.0 | 106.5±12.0 |
| BMI | 32.0±0.4 | 34.5±4.4 | 37.8±2.4 |
| Main complications | Chronic obstructive pulmonary disease, obesity, reflux esophagitis, chronic superficial gastritis, duodenal bulb ulcers, hyperlipidemia | Hyperinsulinemia, hyperlipidemia, hyperuricemia, obesity, sleep apnea syndrome | Type 2 diabetes, hyperinsulinemia, hyperlipidemia, sleep apnea hypopnea syndrome, obesity |
| The intervention | As a liver transplantation donor or bariatric surgery | As a liver transplantation donor or bariatric surgery | bariatric surgery |
| Data are shown as the mean ± SD.  Abbreviations: NAFL, nonalcoholic fatty liver; NASH, nonalcoholic steatohepatitis; BMI, body mass index; | | | |

**Supplemental Table S2. Antibodies for Western blot analysis**

| **Antibody** | **Cat No** | **Manufacturer** |
| --- | --- | --- |
| β-ACTIN | AC026 (1:50000) | ABclonal |
| KDM1A  H3K4ME2  H3 | A1156 (1:1000)  PTM-612 (1:2000)  PTM-1002 (1:2000） | ABclonal  PTM BIO  PTM BIO |

**Supplemental Table S3. Primer sequences for RT-qPCR.**

| **Gene** | **Species** | **Forward primer (5’-3’)** | **Reverse primer (5’-3’)** |
| --- | --- | --- | --- |
| ***CCL2*** | **Human** | CAGCCAGATGCAATCAATGCC | TGGAATCCTGAACCCACTTCT |
| ***CXCL10*** | **Human** | GTGGCATTCAAGGAGTACCTC | TGATGGCCTTCGATTCTGGATT |
| ***TNFα*** | **Human** | TGGCGTGGAGCTGAGAGATA | TGATGGCAGAGAGGAGGTTG |
| ***MCP1*** | **Human** | ATAGCAGCCACCTTCATTCCC | CAGCTTCTTTGGGACACTTGC |
| ***CD36*** | **Human** | TGCAAAGAAGGGAGACCTGTG | GTTGACCTGCAGCCGTTTTG |
| ***SCD1*** | **Human** | TCATAATTCCCGACGTGGCT | CCCAGAAATACCAGGGCACA |
| ***FASN*** | **Human** | ACAGCGGGGAATGGGTACT | GACTGGTACAACGAGCGGAT |
| ***ACCα*** | **Human** | TCACACCTGAAGACCTTAAAGCC | AGCCCACACTGCTTGTACTG |
| ***C/EBPβ*** | **Human** | AAGCACAGCGACGAGTACAA | GTGAGCTCCAGGACCTTGTG |
| ***KDM1A*** | **Human** | AGCGTCATGGTCTTATCAACTTC | ACTCGTCCACCCACACGAT |
| ***β-ACTIN*** | **Human** | CATGTACGTTGCTATCCAGGC | CTCCTTAATGTCACGCACGAT |
| ***Tfrc*** | **Mouse** | TAGGCCGCGGGTTCGAG | TGCTACAAGGGAGTACCCCGA |
| ***Il-1β*** | **Mouse** | CCGTGGACCTTCCAGGATGA | GGGAACGTCACACACCAGCA |
| ***Mcp1*** | **Mouse** | TACAAGAGGATCACCAGCAGC | ACCTTAGGGCAGATGCAGTT |
| ***Tnfα*** | **Mouse** | CATCTTCTCAAAATTCGAGTGACAA | TGGGAGTAGACAAGGTACAACCC |
| ***Cd36*** | **Mouse** | GACTGGGACCATTGGTGATGA | AAGGCCATCTCTACCATGCC |
| ***G6pase*** | **Mouse** | CGACTCGCTATCTCCAAGTGA | GGGCGTTGTCCAAACAGAAT |
| ***Scd1*** | **Mouse** | TCTTCCTTATCATTGCCAACACCA | GCGTTGAGCACCAGAGTGTATCG |
| ***Plin2*** | **Mouse** | GCAACTTGCATTTGTCCGGT | CTCTCATCACCACGCTCTGT |
| ***Pparγ*** | **Mouse** | ATTCTGGCCCACCAACTTCGG | TGGAAGCCTGATGCTTTATCCCCA |
| ***Fatp1*** | **Mouse** | TGCACAGCAGGTACTACCGCAT | TGCGCAGTACCACCGTCAAC |
| ***Fabp1*** | **Mouse** | TGGTCCGCAATGAGTTCACCCT | CCAGCTTGACGACTGCCTTGACTT |
| ***Cpt-1α*** | **Mouse** | AGGACCCTGAGGCATCTATT | ATGACCTCCTGGCATTCTCC |
| ***Acox-1*** | **Mouse** | GTCTCCGTCATGAATCCCGA | TGCGATGCCAAATTCCCTCA |
| ***Mcad*** | **Mouse** | GAAGCCACGAAGTATGCCCT | TAGTAAGTGTTCCGGCGACC |
| ***Pepck1*** | **Mouse** | TGCCCCAGGCAGTGAGGAAGTT | GTCAGTGAGAGCCAGCCAACAGT |
| ***Pgd*** | **Mouse** | TTGACAACTGCCAGGACTCC | GAGCCTGGATGAGGTTTGCT |
| ***G6pase*** | **Mouse** | CGACTCGCTATCTCCAAGTGA | GGGCGTTGTCCAAACAGAAT |
| ***Fbpase*** | **Mouse** | AGTCGTCCTACGCTACCTGTG | GGGGATCGAAACAGACAACAT |
| ***Kdm1a*** | **Mouse** | ATGGTGCTCTGTTGAGTGGG | ATACTTGGGGACTGCTGTGC |
| ***β-Actin*** | **Mouse** | GTGACGTTGACATCCGTAAAGA | GCCGGACTCATCGTACTCC |
| ***Kdm1a*** | **Monkey** | CACCAGCCGTTCAGTTTGTG | GCCAACATGCCCGAACAAAT |
| ***β-Actin*** | **Monkey** | AACCTTCCTTCCTGGGCATG | AATGCCAGGGTACATGGTGG |

**Supplemental Table S4. Primer sequences for molecular cloning.**

| **Gene** | **Forward primer (5’-3’)** | **Reverse primer (5’-3’)** |
| --- | --- | --- |
| Human-*KDM1A* | TCGGGTTTAAACGGATCCATGTTATCTGGGAAGAAGGCGG | GGGCCCTCTAGACTCGAGTCACATGCTTGGGGACTGCT |
| Human-*KDM1A*-mutant | GCAACCTTAACGCAGTGGTGTTGT | ACAACACCACTGCGTTAAGGTTGC |
| Human-*HNF4α* | TCGGGTTTAAACGGATCCATGCGACTCTCCAAAACCCTCG | GGGCCCTCTAGACTCGAGGATAACTTCCTGCTTGGTGATGGTCG |
| Human-*C/EBP-α* | TCGGGTTTAAACGGATCCATGGAGTCGGCCGACTTCT | GGGCCCTCTAGACTCGAGCGCGCAGTTGCCCATGG |
| Human-*C/EBP-β* | TCGGGTTTAAACGGATCCATGCAACGCCTGGTGGC | GGGCCCTCTAGACTCGAGGCAGTGGCCGGAGGAGG |
| Human-*KDM1A*-promotor LUC | TAGATCGCAGATCTCGAGGGGACAAAAAGGGTCGGAGACAC | TACCGAGCTCTTACGCGTAATTTAGCTCCCAAGCAGCACC |
| Human-sh*C/EBP-β* | CCGGCCCGTGGTGTTATTTAAAGAACTCGAGTTCTTTAAATAACACCACGGGTTTTTG | AATTCAAAAACCCGTGGTGTTATTTAAAGAACTCGAGTTCTTTAAATAACACCACGGG |


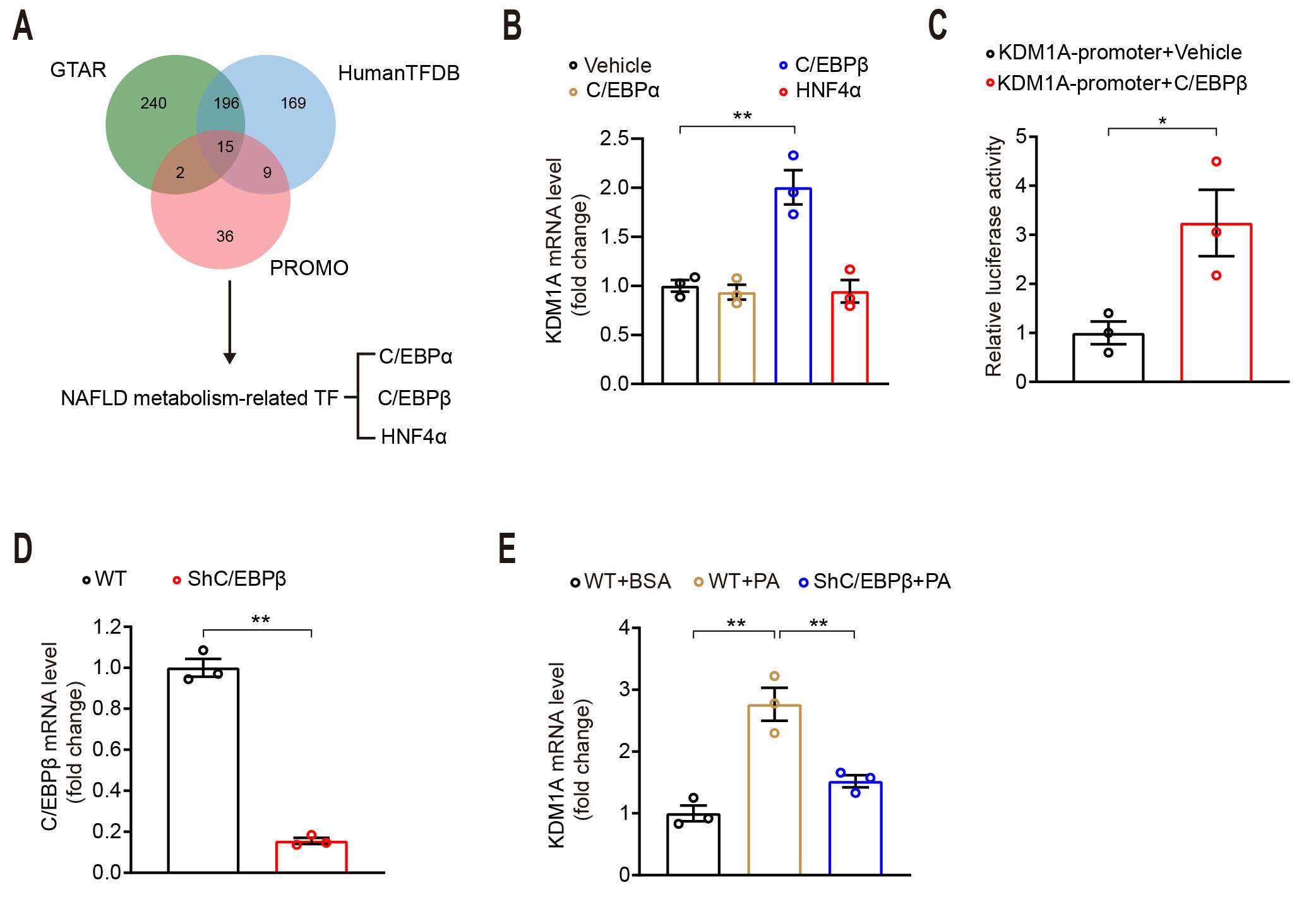
 **Supplemental Figure S1.** *C/EBPβ* is the regulator upstream of *KDM1A* (A) Venn diagram of the predicted transcription factors. (B) Relative mRNA levels of *KDM1A* in WT Huh7 hepatocytes transfected with Vehicle, *C/EBPα*, *C/EBPβ*, and *HNF4α*. The data shown are representative of three independent experiments. (C) Luciferase assay examining the activity of *KDM1A* promotor in Huh7 hepatocytes transfected with Vehicle or *C/EBPβ*. The data shown are representative of three independent experiments. (D) Relative mRNA levels of *C/EBPβ* in WT Huh7 hepatocytes and Sh*C/EBPβ* Huh7 hepatocytes. The data shown are representative of three independent experiments. (E) Relative mRNA levels of *KDM1A* in WT Huh7 hepatocytes treated with BSA or PA and Sh*C/EBPβ* Huh7 hepatocytes treated with PA. The data shown are representative of three independent experiments. Values are presented as mean ± SD. **P* < 0.05, ***P* < 0.01, n.s., not significant; One-way ANOVA statistics was applied in (B, E). Student's t-test was applied in (C, D).


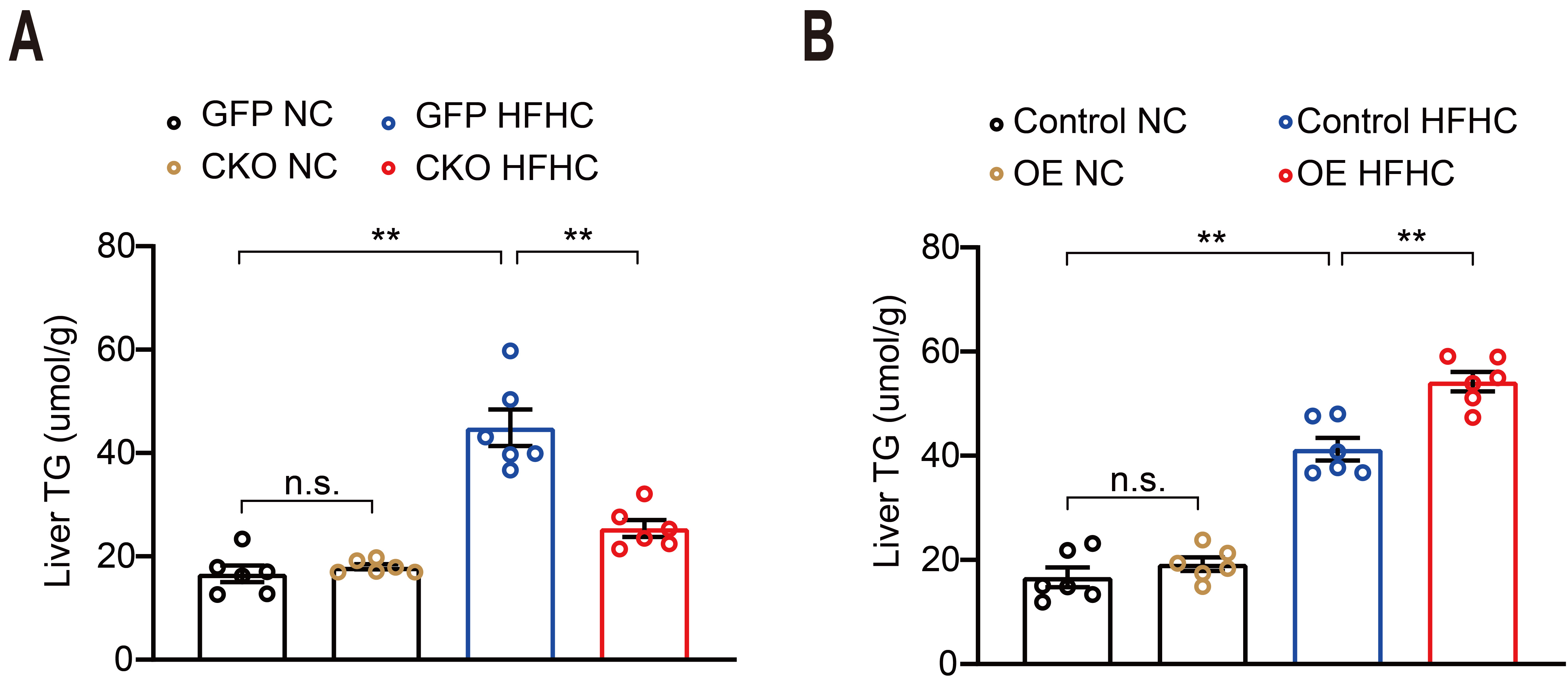


**Supplemental Figure S2.** TG levels of the liver in *Kdm1a* CKO or OE groups**.** (A) Liver TG levels were measured in *Kdm1a*-CKO mice and WT control mice after NC or HFHC consumption for 16 weeks, n = 6 mice per group. (B) Liver TG levels were measured in *Kdm1a*-OE mice and WT control mice after NC or HFHC consumption for 16 weeks, n = 6 mice per group. Values are presented as mean ± SD. **P* < 0.05, ***P* < 0.01, n.s., not significant; One-way ANOVA statistics was applied.

**
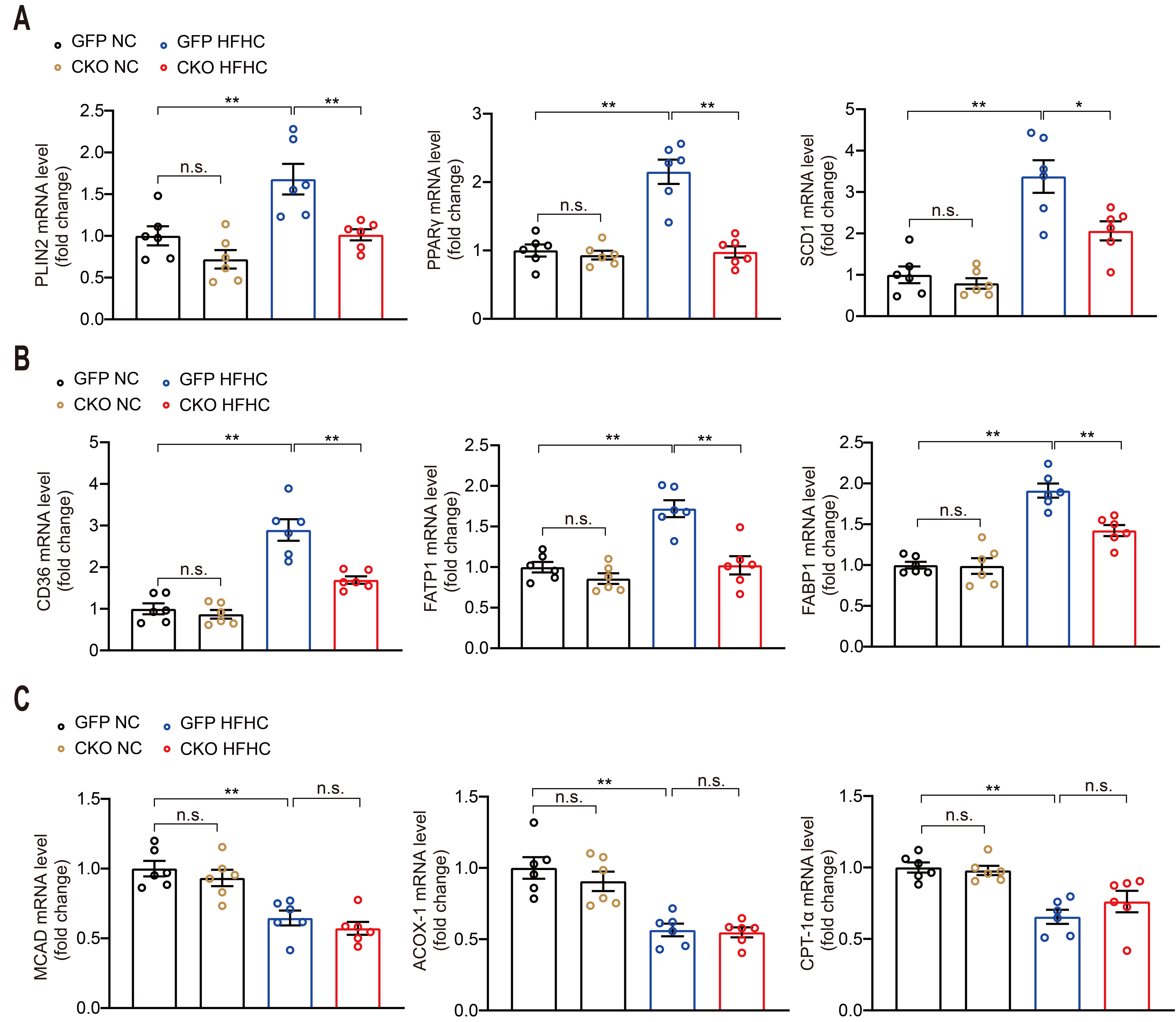
**

**Supplemental Figure S3.** Deletion of *Kdm1a* improves lipid metabolism through downregulation of genes related to lipid biosynthesis and uptake. (A) Relative mRNA levels of genes related to lipid biosynthesis in the livers of NC or HFHC diet-fed mice with or without *Kdm1a* CKO. n=6 mice per group. (B) Relative mRNA levels of genes related to lipid uptake in the livers of NC or HFHC diet-fed mice with or without *Kdm1a* CKO. n=6 mice per group. (C) Relative mRNA levels of genes related to lipid degradation in the livers of NC or HFHC diet-fed mice with or without *Kdm1a* CKO. n=6 mice per group. Values are presented as mean ± SD. **P* < 0.05, ***P* < 0.01, n.s., not significant; One-way ANOVA statistics was applied.


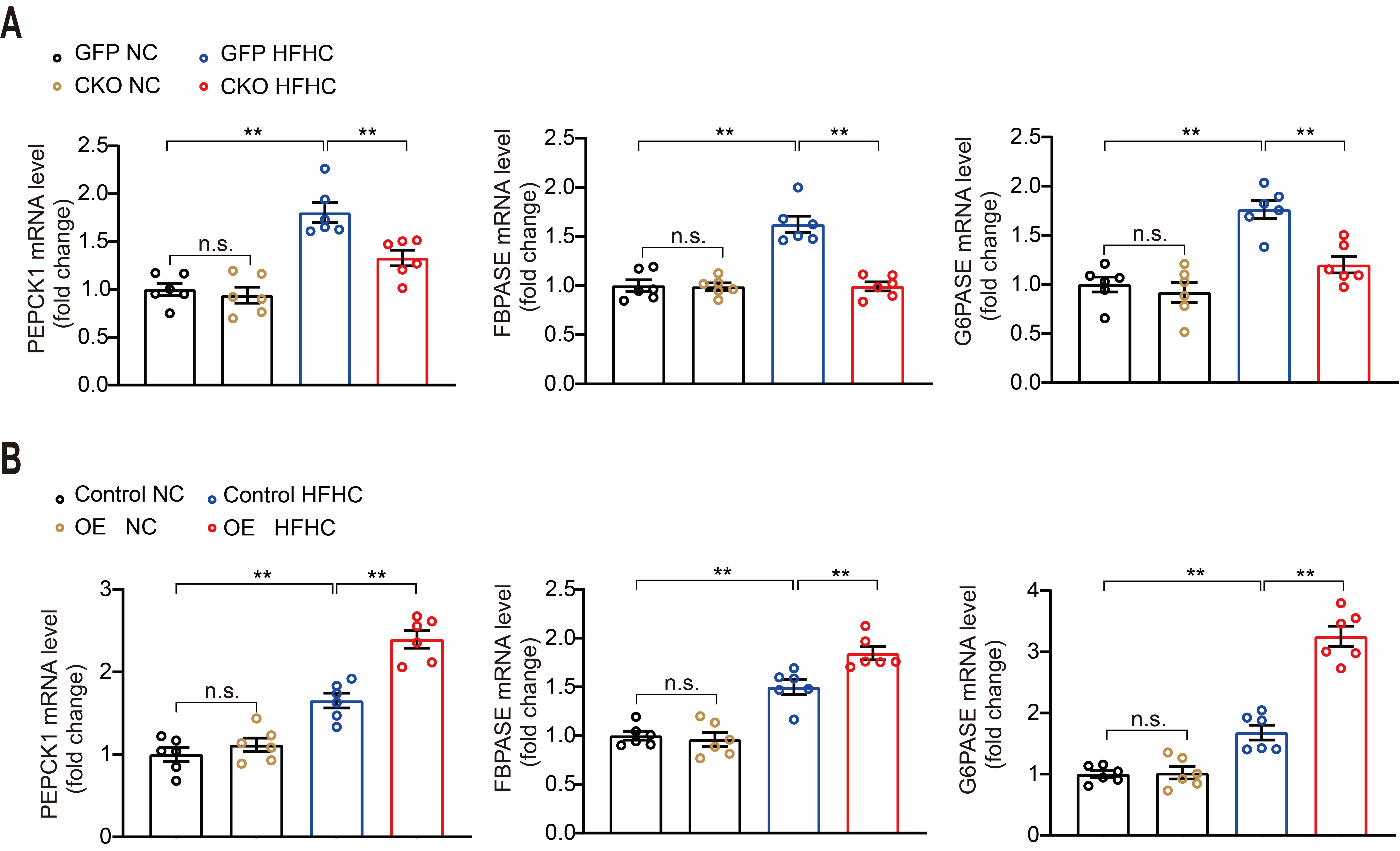


**Supplemental Figure S4.** The expression of genes related to glucose metabolism in *Kdm1a* CKO or OE groups (A) Relative mRNA levels of genes related to gluconeogenesis in the livers of NC or HFHC diet-fed mice with or without *Kdm1a* CKO. n=6 mice per group. (B) Relative mRNA levels of genes related to gluconeogenesis in the livers of NC or HFHC diet-fed mice with or without *Kdm1a* OE. n=6 mice per group. Values are presented as mean ± SD. **P* < 0.05, ***P* < 0.01, n.s., not significant; One-way ANOVA statistics was applied.


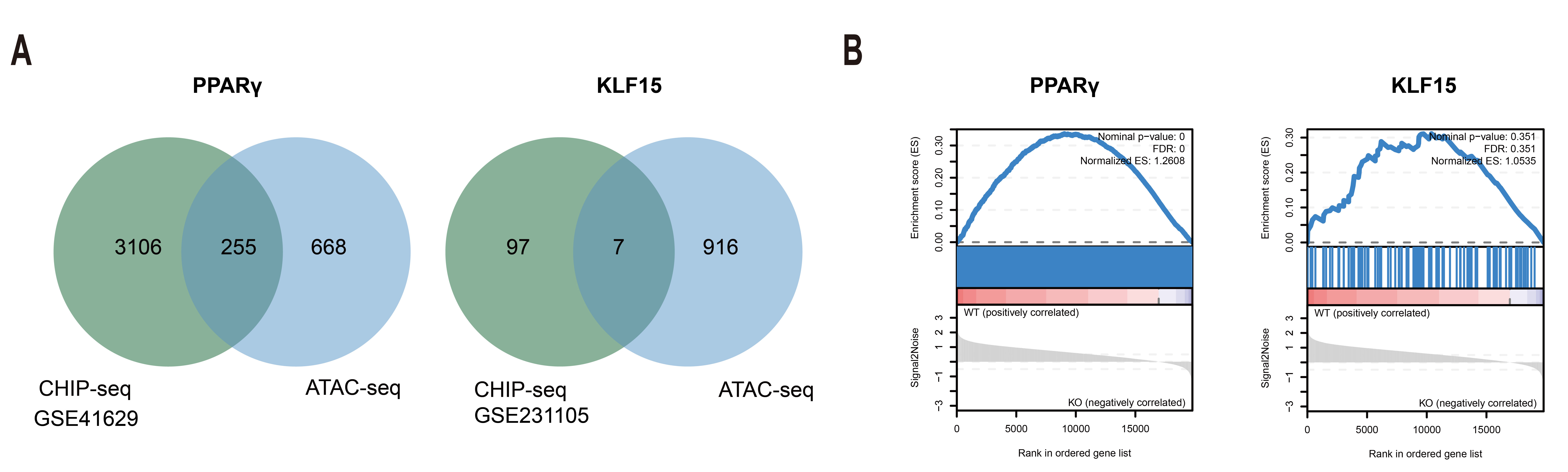


**Supplemental Figure S5.** Evaluation of transcription factors (A) Venn plot showing intersection of genes (promoter and distal region) annotated by peaks in the public CHIP-seq databases of transcription factor *Pparγ* or *Klf15* with differential peaks in ATAC-seq. (B) GSEA analysis of the expression levels of peak annotated genes (promoter and distal regions) in public CHIP-seq databases and the gene expression levels of peak annotated genes in ATAC-seq.
